# Multimodal X-ray imaging of grain-level properties and performance in a polycrystalline solar cell

**DOI:** 10.1107/S1600577519003606

**Published:** 2019-05-14

**Authors:** A. Ulvestad, S. O. Hruszkewycz, M. V. Holt, M. O. Hill, I. Calvo-Almazán, S. Maddali, X. Huang, H. Yan, E. Nazaretski, Y. S. Chu, L. J. Lauhon, N. Rodkey, M. I. Bertoni, M. E. Stuckelberger

**Affiliations:** a Materials Science Division, Argonne National Laboratory, Argonne, Illinois 60439, USA; b Center for Nanoscale Materials, Argonne National Laboratory, Argonne, Illinois 60439, USA; c Department of Materials Science and Engineering, Northwestern University, Evanston, Illinois 60208, USA; d National Synchrotron Light Source II, Brookhaven National Laboratory, Upton, New York 11973, USA; e Electrical, Computer, and Energy Engineering, Arizona State University, Tempe, Arizona 85287, USA; f Photon Science, Deutsches Elektronen-Synchrotron, 22607 Hamburg, Germany

**Keywords:** solar cell materials, scanning nanodiffraction, X-ray-beam-induced current, multimodal characterization

## Abstract

The factors limiting the performance of alternative polycrystalline solar cells as compared with their single-crystal counterparts are not fully understood, but are thought to originate from structural and chemical heterogeneities at various length scales. Here, it is demonstrated that multimodal focused nanobeam X-ray microscopy can be used to reveal multiple aspects of the problem in a single measurement by mapping chemical makeup, lattice structure and charge collection efficiency simultaneously in a working solar cell. This approach was applied to micrometre-sized individual grains in a Cu(In,Ga)Se_2_ polycrystalline film packaged in a working device. It was found that, near grain boundaries, collection efficiency is increased, and that in these regions the lattice parameter of the material is expanded. These observations are discussed in terms of possible physical models and future experiments.

## Abstract 2

A multimodal *in situ* nanofocused X-ray microscopy approach is demonstrated and applied to a working polycrystalline thin film solar cell that revealed chemical, structural and electronic heterogeneity from a single measurement.

## 1. Introduction   

As the worldwide use and adoption of photovoltaic (PV) modules for electricity generation continue to increase (Haegel *et al.*, 2017 ▸), new challenges arise. For example, the energy required to fabricate the crystalline silicon solar cells that dominate today’s market is no longer negligible. Furthermore, this technology cannot be scaled indefinitely due to the complexity of the manufacturing processes and elemental scarcity associated with these architectures. Such considerations motivate the development of alternative PV platforms.

Among these promising alternatives, thin-film solar cells based on direct-bandgap semiconductors require two orders of magnitude less absorber material than Si. This directly translates into shorter energy-payback times (Wild-Scholten, 2013 ▸), and deposition methods that are more readily compatible with industry-friendly roll-to-roll processes, which make better use of expensive machine time and enable constant performance due to the lack of stop-and-go batch processing. For this purpose, flexible substrates such as stainless steel or polyimide foils that are compatible with high-temperature film deposition processes have been adopted for industrial applications. Although the gap between the conversion efficiency of thin-film solar modules and silicon modules is closing, commercial thin-film modules lag behind their crystalline silicon counterparts in efficiency (Green *et al.*, 2018 ▸). To a large extent, this is caused by inhomogeneities at different length scales: at the sub-millimetre level, performance variations are often caused by processing imperfections, whereas performance variations at the sub-micrometre level are often caused by heterogeneity in the nano- and microcrystalline structure and composition. In particular, grain boundaries with enhanced defect concentrations serve as recombination centers, and the composition gradients that surround them can lead to band bending and electronic variations. Such variations in potential inevitably reduce the maximum achievable solar cell voltage (Siebentritt, 2011 ▸).

Scanning X-ray microscopy with nanofocused X-rays offers the possibility to characterize solar cells across these relevant length scales with sensitivity to local structural, chemical and electrical performance. This is possible due to the fact that, with specialized instrumentation, measurements of X-ray fluorescence (XRF), X-ray-beam-induced current (XBIC) (Vyvenko *et al.*, 2002 ▸; Stuckelberger, West *et al.*, 2017 ▸), X-ray-beam-induced voltage (XBIV) (Stuckelberger *et al.*, 2018 ▸; Stuckelberger, Nietzold, West *et al.*, 2017 ▸), and lattice strain and tilt (via Bragg peak analysis) can be made simultaneously within the nanoscale volume illuminated by the beam. This allows heterogeneities in these quantities to be mapped and correlated within a single grain and near the grain boundaries of a functioning thin-film PV device (West *et al.*, 2017 ▸; Buonassisi *et al.*, 2005 ▸; Stuckelberger, Nietzold, Hall *et al.*, 2017 ▸). Compared with previous studies, this work integrates Bragg diffraction as a new mode of structural contrast, expanding upon and broadening previous capabilities. Applying this approach to a commercially produced thin-film solar cell with a Cu(In,Ga)Se_2_ (CIGS) absorber on a stainless steel substrate, we explore correlations between heterogeneous structure, composition and performance at sub-grain resolution, paving the way towards a more complete picture of the local structure–property relationship of CIGS specifically, and the class of polycrystalline thin-film PV materials more generally.

## 2. Experimental   

The X-ray measurements were performed at the 3-ID HXN beamline (Nazaretski *et al.*, 2017 ▸; Yan *et al.*, 2018 ▸) of the NSLS-II synchrotron using a ∼100 nm-diameter FWHM (full width at half-maximum) X-ray beam (10.4 keV energy) focused with a Fresnel zone plate. The sample used in this work was a fully operational industrial solar cell with a thin-film Cu(In,Ga)Se_2_ absorber, 1.7 µm in thickness, manufactured by MiaSolé HiTech Corporation on stainless steel (Macki *et al.*, 2013 ▸; Farshchi *et al.*, 2016 ▸). In the CIGS film, the nominal ratio of Ga composition expressed in terms of the gallium-to-gallium-plus-indium (GGI) ratio was 0.3.

**Figure 1:**
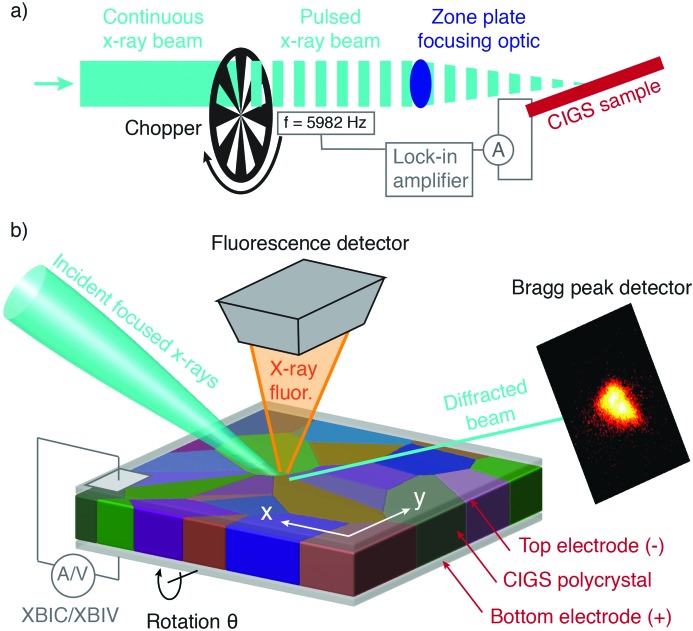
A schematic of the experimental setup. Panel (*a*) shows the integration of an optical chopper and lock-in amplifier into the X-ray beamline that enables position-resolved XBIC and XBIV measurements. Panel (*b*) shows more details of the sample–beam interaction area: a PV device containing an active layer of polycrystalline CIGS between top and bottom electrodes was illuminated with a nanofocused X-ray beam. The orientation of the beam was such that a 112 Bragg peak could be observed from grains that were favorably oriented. Raster scans of the sample were performed by displacing the sample in the plane of the film in ∼100 nm steps. During the raster scan simultaneous measurements were performed of the local Bragg diffraction, the emitted X-ray fluorescence spectrum, and either the XBIC or XBIV. To measure 3D Bragg peak information, raster scans were repeated at different incident angles by varying the sample angle (along the θ rotation axis in the figure).

The measurement of XBIC and XBIV was enabled by incorporating an encoded rotating X-ray chopper into the optical path of the X-ray beam. The chopper periodically blocked the beam at 5982 Hz in order to produce a pulsed X-ray beam at the sample from which a differential ‘beam-on’ versus ‘beam-off’ PV electrical response could be measured. This arrangement is shown schematically in Fig. 1 ▸. The current or voltage output of the solar cell was input to a lock-in amplifier tuned to isolate electrical signal from the sample at the frequency of the upstream chopper, in a manner analogous to lock-in-amplified PV quantum efficiency measurements. Thus, the output of the lock-in amplifier enabled clean XBIC and XBIV measurements at each X-ray scan position. More details as to this method of measuring XBIC/XBIV are described in the work of Stuckelberger, West *et al.* (2017 ▸) and Stuckelberger, Nietzold, West *et al.* (2017 ▸).

The surface of the sample was positioned in the focal plane of the lens and in the center of rotation of the goniometer, allowing the same area of the sample to be scanned at a series of incident-beam angles with the focused beam (see Fig. 1 ▸). In order to measure Bragg diffraction, the angle between the sample surface and the incident beam was set to ∼10°, and a pixel array detector was positioned half a metre from the sample, offset from the direct beam by 21° in order to satisfy the 112 Bragg condition of crystalline CIGS. This detector position was achieved by combined angular detector arm motions of 16° in the film-normal plane parallel to the incident beam and 10° normal to this plane. The shallow incident angle was required due to several experimental constraints, including the fact that the substrate below the film was not X-ray transparent, and that in that case steep incident angles occlude the fixed fluorescence detector. In this geometry, only a small subset of grains were expected to diffract due to the diversity of grain orientation in the sample. This orientational polydispersity allowed for a clean measurement of diffraction from individual grains, without any scattering signal from neighboring grains that are oriented far from their diffraction conditions. This scanning geometry also allowed efficient collection of XRF, as the illuminated surface of the sample faced a fluorescence detector that subtended a large solid angle of the sample, enabling compositional analysis at every scan point together with electrical measurements (XBIC or XBIV). Candidate grains were identified with a coarse 20 µm × 20 µm overview scan from which several suitable diffracting grains were chosen for study.

The five grains chosen for high-resolution analysis had an average diameter of ∼2 µm, typical of the active material layer. The measurements of each grain involved a ∼4 µm × 4 µm raster scan of the sample surface with step sizes of 50 and 250 nm in the *x*, *y* in-plane directions of the film. (In the synchrotron experimental laboratory frame, the sample was mounted such that *y* could be scanned by translating the sample vertically, and the *x* direction could be scanned via horizontal translation.) These step sizes were chosen to correspond to half the size of the beam footprint on the sample surface in the two directions, which was 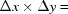 100 nm × 575 nm due to the shallow incident angle of the beam. The first raster map of a given grain was done to measure baseline XBIV, after which the electronics were switched to monitor XBIC for all subsequent maps. Full 3D reciprocal-space characterization of the 112 Bragg peak of a given grain as a function of position required 11 sample raster scan measurements made at a series of angles spanning ±0.5° about the Bragg peak maximum in 0.1° increments. Such an angular scan is known as a rocking curve and is designed to bracket the angular breadth of the Bragg peak in 3D, as is commonly done in X-ray nanodiffraction experiments (Holt *et al.*, 2013 ▸).

Positional registration of this series of maps was ensured by correlating the maps of Ga and Cu *K*-edge fluorescence. The Ga and Cu fluorescence maps were generated by fitting the full fluorescence spectrum at each position and reporting the integrated area of the fitted Ga *K* and Cu *K* emission lines. At the X-ray energy used in this study, In *L*-edge fluorescence was excited in the sample and also detected in the fluorescence detector. However, the signal-to-noise ratio of the resulting In fluorescence spatial maps was poor due to the fact that the X-ray beam energy was far from the In *L*-edge resonance energy (∼4 keV), and we do not consider these noisy data in our analysis. One complete data set for a single grain took ∼100 min to complete. We established that beam-induced damage in the CIGS was minimal during the measurement time by noting that the XBIC maps remain mostly unchanged. Nevertheless, in order to represent the minimally dosed case, the XBIC maps, Ga *K* maps and Cu *K* maps analyzed below are those from the first raster scan of the rocking curve. The full set of raster maps as a function of position and rocking angle were used to determine the local lattice structure.

From the Bragg reciprocal-space maps at each position, the relative compressive/tensile strain was extracted by determining the radial angle of the maximum of the Bragg peak, from which the length of the reciprocal-lattice vector was determined (Hill *et al.*, 2018 ▸). Variations in the length of the reciprocal-lattice vector relative to the mean for a given grain were converted to units of relative uniaxial strain normal to the (112) lattice planes, 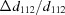, providing a local measure of strain variability. Similarly, the azimuthal angle of the Bragg peak (corresponding to the angular position of the Bragg peak along the Debye–Scherrer ring) was converted to relative deviations in lattice tilt.

## 3. Results and discussion   

**Figure 2:**
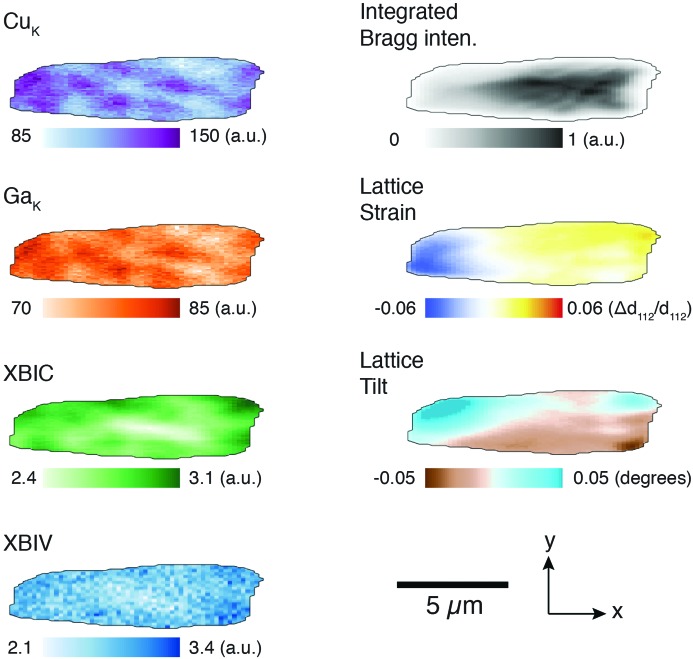
Scanning nanofocused X-ray beam measurements of one of the five grains in this study are shown. These include elemental maps obtained through analysis of fluorescence spectra (Cu *K* and Ga *K* maps), monitoring of local cell performance (XBIC, XBIV), and analysis of the 3D Bragg peak (integrated intensity, lattice strain and lattice tilt) measured at each beam scan position.

Fig. 2 ▸ shows a typical grain in our study, plotted in terms of the modes of elemental, structural and electrical contrast extracted from the scanning probe X-ray measurement. The edges of this grain (and all other grains) were determined using a 10%-of-maximum contour of the integrated Bragg intensity map, and pixels above this threshold were considered for analysis. We note that the image of the grain is elongated in the *x* direction, corresponding to the long footprint direction of the beam on the sample surface due to the shallow incidence angle. This footprint produces an asymmetric resolution function and complicates analysis, as we discuss below, but is necessary given the constraints of the Bragg condition. We also note that 68% of the electron–hole pairs that contribute to XBIC signal is estimated to be generated within  100 nm from the central beam path at an X-ray energy of 10.4 keV, matching well with the choice of scan step size. The penetration depth along the beam path, within which 68% of the incident photons are absorbed, is greater than 10 µm. Therefore, XBIC, XRF and Bragg diffraction signal are generated along the entire beam path in the sample. Thus, the maps enable two types of analysis. Firstly, we can make observations directly from the spatial maps, and for this we focus on the XBIC and lattice strain modes of contrast. Secondly, we can quantify pixel-wise correlations by calculating a correlation matrix. We discuss these analyses below and the conclusions we draw from them.

**Figure 3:**
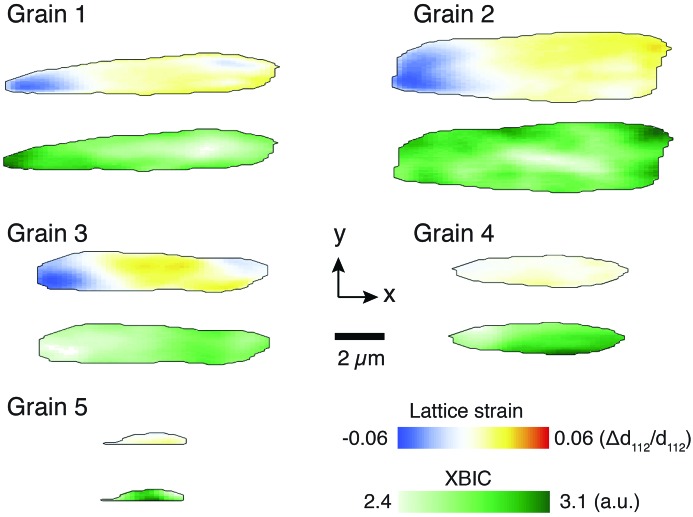
All five grains measured in this study, shown in terms of (112) lattice strain and XBIC. The larger grains (1–3) extend from the top to the bottom of the CIGS film, while the smaller grains (4–5) do not.

Maps of XBIC and strain are shown for the five grains measured in this study in Fig. 3 ▸. Grains 1–3 are larger, and their extent in the *x* direction indicates that they span from the bottom to the top of the CIGS layer. For a grain that is 2 µm wide in the *x* dimension and that spans the entire film height of 1.7 µm illuminated at a 10° incident angle, we expect the images in Fig. 3 ▸ to have an *x*-extent of 11.6 µm. Grains 1–3 indeed show such characteristic sizes. As a result, these grains display a distinct *x*-dependent strain profile that is expected due to the depth-dependent gradient in GGI ratio. This profile is visible in these images due to the fact that, with a shallow incidence angle, the beam first encounters the buried bottom edge of the grain in the left edge of the image and progresses to illuminate the top edge of the grain in the right side of the image. Spatially dependent information can also be gleaned by considering the XBIC maps of the larger grains. Especially in grains 1 and 2, higher XBIC signal was observed near the edges of the grain, which suggests that charge collection efficiency may be higher near grain boundaries, in agreement with other studies involving high-efficiency CIGS solar cells (West *et al.*, 2017 ▸). These types of visual trends are not as readily apparent with the smaller grains (4, 5) or with the other modes of contrast available in this measurement; however, we can collate all the information available from all grains via correlation coefficient analysis.

**Figure 4:**
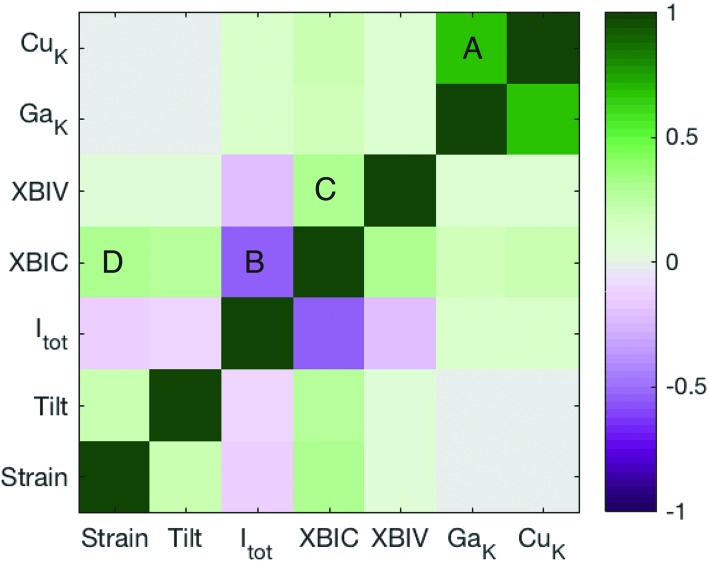
Correlation matrix determined from the 2D focused X-ray beam raster maps of structure, composition and electronic properties. A single correlation matrix was calculated using all pixels within all five grains in this study.

A correlation coefficient matrix of the modes of contrast across all grains is shown in Fig. 4 ▸. In order to exclude correlations stemming from strong depth-dependent effects such as GGI gradient discussed above and X-ray self-absorption, we composed this correlation matrix so as to exclude variations in the *x* direction. This was done by calculating Pearson correlation coefficient matrices on a column-by-column basis (along the *y* direction) for all columns of pixels in all grains. These column-wise correlation matrices were summed and weighted by the number of pixels in each column. The result in Fig. 4 ▸ therefore represents correlation of electronic, structural and compositional features along the higher-resolution *y* scan direction.

We find that several features are correlated and provide insight as to the performance of the solar cell. The strongest salient correlations that will be discussed here are labeled with letters in Fig. 4 ▸. Firstly, a strong positive correlation was observed between Cu *K*-edge and Ga *K*-edge fluorescence (point *A* in Fig. 4 ▸). This is simply related to spatial variations in the overall thickness in the film (peaking at grain centers) expected during synthesis of this material. More relevant insight can be gained from points *B* and *C*. We observed a negative correlation between XBIC and the angle-integrated Bragg peak intensity (point *B*). Because integrated Bragg peak intensity decreases near the grain edges due to decreasing interaction volume with the grain, this negative correlation reflects our earlier observation based on assessment of scans that XBIC tends to be higher near grain boundaries. Point *C* further corroborates the earlier stated hypothesis that higher charge collection efficiency is present near grain boundaries. This is because a positive correlation between XBIC and XBIV indicates that spatial variations in internal quantum efficiency dominate the observed trends rather than spatial bandgap variations (Stuckelberger, Nietzold, West *et al.*, 2017 ▸).

Finally, we consider point *D* in Fig. 4 ▸, which indicates a weak positive correlation between XBIC and positive uniaxial strain [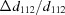]. This correlation, though subject to limitations discussed in the next paragraph, indicates that regions of higher charge collection efficiency have a larger local lattice parameter. We put forth two possible physical pictures that are consistent with this correlation, but that require further corroboration. Firstly, the larger lattice parameter and enhanced XBIC may be due to the segregation of indium or copper to the grain boundaries. Another possibility is that lateral lattice parameter changes are due to residual stresses from the synthesis process that influence electrical properties rather than compositional fluctuations. These hypotheses can both account for the connection between strain variations and local electrical properties, but require further measurements with specific modifications for definitive corroboration, as discussed below.

A few limitations of our current measurement are noteworthy and suggest improvements for future experiments. First, the shallow incidence angle of the beam weakens the correlation of XRF and XBIC to those quantities derived from the Bragg peak because of the very long illumination pathway of the beam compared with the grain size in this material. This creates a disparity in the origin of the signal of the structural maps, which originates from Bragg diffraction of a single grain in the beam path, and the XRF/XBIC/XBIV maps, that are integrated from the entire beam path in the sample, including signal from adjacent grains. Thus, we expect a degree of decorrelation of the signals that could be mitigated if an angle of incidence closer to 90° was adopted, as was done in previous XBIC/XRF studies that did not include Bragg nanodiffraction (Stuckelberger, West *et al.*, 2017 ▸). Secondly, in our experiment we were limited to monitoring Bragg peaks in a reflection geometry, making it highly impractical to measure more than one Bragg peak per grain.

Both of these issues could potentially be resolved by a modification of the solar cell geometry to allow measurement of Bragg peaks in a Laue geometry through the bottom electrode and support substrate. This could enable the measurement of multiple Bragg peaks needed to resolve more than one component of the local strain tensor while maintaining a near-normal beam incidence angle. Additionally, one can envision adopting a much higher X-ray energy that would allow the excitation of indium *K*-edge fluorescence (27.9 keV) in order to quantify the degree of indium segregation near grain boundaries. Finally, in such a proposed geometry, the spatial resolution could be significantly improved either by employing more tightly focusing X-ray multi-layer Laue lenses (Xu *et al.*, 2017 ▸; Yan *et al.*, 2018 ▸) that work efficiently at ∼30 keV X-ray energies, or by utilizing coherent diffraction imaging approaches that enable sub-beam-sized resolution in 3D (Hill *et al.*, 2018 ▸; Hruszkewycz *et al.*, 2017 ▸). Such experiments are now being designed to build upon this work.

## 4. Conclusions   

Our correlative X-ray microscopy approach elucidates some of the factors that play a role in the performance of a working solar cell containing Cu(In,Ga)Se_2_, a promising alternative polycrystalline solar cell absorber material. Namely, through correlation analysis of structural, electronic and compositional maps of individual micrometre-scale grains obtained with scanning nanofocused synchrotron X-rays, we find that near grain boundaries, collection efficiency is increased, and that in these regions the lattice parameter of the material is expanded. Both of these observations can potentially be explained by indium or copper segregation at grain boundaries or by remnant stresses from the synthesis process, but await further exploration with improved experiments. Generally, these results fit with the literature consensus that controlling structure and composition gradients near grain boundaries in polycrystalline absorber materials, though difficult to achieve, should be considered for improved performance. Beyond shedding light on the local structure/property relations that dictate performance in CIGS solar cells, this work demonstrates the utility of multi-modal scanning nanofocused X-ray measurements for other alternative solar cell materials, and for nanostructured multi-component electronic materials in general.
